# Chromosome 1q21 abnormalities in multiple myeloma

**DOI:** 10.1038/s41408-021-00474-8

**Published:** 2021-04-29

**Authors:** Timothy M. Schmidt, Rafael Fonseca, Saad Z. Usmani

**Affiliations:** 1 University of Wisconsin Carbone Cancer Center, Madison, WI USA; 2 Department of Hematology, Mayo Clinic, Scottsdale, AZ USA; 3 Plasma Cell Disorders Division, Levine Cancer Institute/Atrium Health, Charlotte, NC USA

**Keywords:** Cancer genomics, Myeloma, Myeloma

## Abstract

Gain of chromosome 1q (+1q) is one of the most common recurrent cytogenetic abnormalities in multiple myeloma (MM), occurring in approximately 40% of newly diagnosed cases. Although it is often considered a poor prognostic marker in MM, +1q has not been uniformly adopted as a high-risk cytogenetic abnormality in guidelines. Controversy exists regarding the importance of copy number, as well as whether +1q is itself a driver of poor outcomes or merely a common passenger genetic abnormality in biologically unstable disease. Although the identification of a clear pathogenic mechanism from +1q remains elusive, many genes at the 1q21 locus have been proposed to cause early progression and resistance to anti-myeloma therapy. The plethora of potential drivers suggests that +1q is not only a causative factor or poor outcomes in MM but may be targetable and/or predictive of response to novel therapies. This review will summarize our current understanding of the pathogenesis of +1q in plasma cell neoplasms, the impact of 1q copy number, identify potential genetic drivers of poor outcomes within this subset, and attempt to clarify its clinical significance and implications for the management of patients with multiple myeloma.

## Introduction

Multiple myeloma (MM) is the second most common hematological malignancy^1^ and fortunately has seen substantial improvement in patient survival over the past two decades^2^. Although MM is characterized by marked biological and genetic heterogeneity, the improvement in outcomes has been driven primarily by treatment regimens that target key components of plasma cell biology rather than the underlying genomic aberrancies^3^. While almost all patients seem to benefit somewhat from novel therapies, many patients still experience an aggressive disease course with early relapse, clinical morbidity, and early mortality. Screening for recurrent cytogenetic abnormalities (CA) remains one of the most powerful means to identify patients who are at higher risk for early progression and death and who may be candidates for investigational approaches to improve survival.

MM is a biologically heterogeneous disease that has been historically been characterized by either having either having extra chromosome copies (or hyperdiploidy) or translocations associated with chromosome 14 where the immunoglobulin heavy chain (IgH) gene resides. Originally described as such using conventional karyotyping in the 1970s, fluorescent in situ hybridization was utilized in the late 1990s to better characterize the disease using this nomenclature. There is a growing recognition that disease biology dictates outcomes in uniformly treated MM patients and trials enriching for specific subgroups that have short survival outcomes with standard of care approaches need to be explored instead of the one-size-fits-all approach.

After discovery that large chromosomal structural changes were common among many cancer types, additional copies of chromosome 1q (+1q) was identified as one of the most common CA on metaphase cytogenetics in MM^4,5^ and other cancers^6–8^. Although abnormalities on karyotype were noted to impact prognosis in MM^9^, the sensitivity and accuracy of karyotyping to identify large chromosomal aberrations remains suboptimal due to the low proliferative rate of plasma cells^10–12^. Fluorescent in situ hybridization (FISH) overcame many of these barriers in MM^13^ and found that copy number alterations were not only common in MM^11,13,14^, but that abnormalities detected by FISH were also highly prognostic of outcomes^15^. Because of its high sensitivity and reproducibility, FISH has become the primary means to identify recurrent CA in MM and has been incorporated into the Revised International Staging System (R-ISS)^16^. By FISH, +1q is identified in approximately 40% of newly diagnosed MM cases^17^.

Despite its frequency, considerable debate remains regarding the prognostic impact of +1q in MM. One major barrier to understanding the impact of 1q abnormalities in MM is the lack of uniformity in reporting and annotation of cytogenetics^18^, and this is even more profound when reporting chromosome 1 abnormalities. This is particularly important because copy number of 1q has emerged as a very important feature of this CA. In general, +1q should be understood as denoting any patient who has at least one extra copy of some portion of chromosome 1q. Probes detecting DNA on chromosome 1 in region 2, band 1 (1q21) are used to detect this abnormality. Among patients with +1q, gain(1q) describes patients with only one additional copy of 1q (3 total copies, commonly reported in a clinical FISH report as “nuc ish 1q21x3”), whereas amp(1q) denotes patients who have amplification of 1q, defined as two or more extra copies (4 or more total copies). Although some labs report “duplication” of 1q or ratios of 1q21:1p32 (CKS1B:CDKN2C) of >1.1, these annotations do not specify copy number and should be avoided. We propose that these common definitions be uniformly applied in reporting chromosome 1q copy number in MM, and this has been highlighted for emphasis in Table 1. The present review summarizes the pathophysiology of 1q abnormalities in MM, compiles key data regarding the prognostic impact of chromosome 1q21 abnormalities in MM, and attempts to clarify clinical trial outcomes and management implications for patients with +1q myeloma.

**Table 1:** Definitions of various chromosome 1 abnormalities.

+1q^†^	Additional copies of any part of the long arm of chromosome 1(1q), irrespective of copy number or segment of DNA that is gained
Gain(1q)^†^	Gain of only 1 extra copy of chromosome 1q (3 total copies)
Amp(1q)^†^	“Amplification” of 1q, with at least 2 extra copies of chromosome 1q (4 or more total copies)
Trisomy 1	Gain of both arms of chromosome 1, including 1p
C1A	“Chromosome 1 abnormality”, including any abnormal signal(s) in 1q and/or 1p

^†^In MM, 1q is frequently expanded to 1q21, a more specific denotation that specifies region 2, band 1 on the long arm of chromosome 1. The FISH probe used for this region is most commonly CKS1B, and is reported as “nuc ish CKS1BxN”, with “N” denoting the number of signals (i.e., copies) seen using the CKS1B probe.

## Pathogenesis

In addition to the prognostic impact of cytogenetics, characterization of recurrent genetic abnormalities has helped aid in our understanding of myelomagenesis and clonal progression. In ~60% of patients, myeloma is characterized by having extra copies of odd numbered chromosomes, known as hyperdiploidy or trisomic MM, and the remaining cases typically involve translocations involving the IgH locus at chromosome 14q32. These events are almost always mutually exclusive^19–21^, but additional genetic changes including chromosome 1 abnormalities often occur as secondary events and can be seen among patients in both groups. The frequency and copy number of +1q seem to increase as the disease progresses from smoldering myeloma to newly diagnosed MM to relapsed/refractory disease^17,22,23^. In nearly all series, +1q is identified at a higher frequency among patients with high-risk IgH translocations, −13, del(1p), and other high-risk clinical features such as hypercalcemia, but it can still be found among patients with standard risk MM including trisomies^24–26^.

Genomic instability appears to be a hallmark of +1q MM. In what has been described as “jumping 1q syndrome” by the University of Arkansas for Medical Sciences Myeloma group (UAMS-MIRT), patients with +1q were noted to have frequent and common breakpoints in the pericentromeric heterochromatin^27,28^, with subsequent translocation of a 1q segment or whole arm to other chromosomes. This unstable chromatin persists after the original event, leading to a repetitive pattern of translocations or segmental duplications that causes the same segment of DNA from 1q to “jump” around the genome, with increasing copy number as genomic instability becomes worse, frequently upon disease progression^29,30^. Trisomy of chromosome 1 is also commonly seen, although it is not clear whether this is associated with a different clinical course compared to “jumping 1q”. Newer genomic techniques have helped to reveal that multiple myeloma is often characterized by complex structural variants. Within CoMMpass, a prospective study examining the impact of genomics on outcomes of >1000 MM patients, it is notable that +1q21 was most commonly found within the context of whole arm gains rather than chromothripsis^31^, consistent with the pathogenesis previously identified as large copy number gains. Chromosome 1 copy number variations (CNV), including both +1q and del(1p) are also frequently seen within the context of hypodiploidy in MM samples, and clustering analysis of individual CNV has shown that +1q does not cluster with gains or loss of 1p, and that these likely occur as separate events within a complex genetic landscape^32^. Ultimately, regardless of the mechanisms underlying the development of +1q, the main question is whether this occurs as a byproduct of high-risk biology and genomic instability, or whether increased expression of genes on extra copies of 1q causes high-risk disease, resulting in resistance to therapy and early progression/death.

## Potential genetic drivers on chromosome 1q

As chromosome 1q is the largest chromosome arm in the human genome, the search for a particular driver of more aggressive disease has identified numerous candidates, but without clear identification of a gene or pathway that is consistently dysregulated among patients with +1q myeloma. As mentioned above, a key reason for this is that +1q occurs as a secondary event, often in conjunction with other high-risk cytogenetic abnormalities and genomic complexity that complicates the search for a main driver of outcomes and response to therapy. Furthermore, there are almost always multiple pathways involved, and although gene expression profiling (GEP) has helped to characterize patterns of gene dysregulation, the search for a precise, targetable gene has been difficult. As will be explored in this section, several key biological pathways, including cell cycle regulators, apoptosis, interactions with the bone marrow microenvironment, and others have been implicated in genetic aberrations found among MM patients with +1q and are potential candidates as key regulators of pathogenesis in +1q myeloma. Interestingly, despite the heterogeneity of genes involved, many of the cellular functions encoded by these genes seem to be inter-related and result in activation the JAK/STAT3 pathway, either directly or as a downstream effect.

The UAMS-MIRT group, through their pioneering of GEP in MM, found that one subset of MM with high risk features was defined by overexpression of genes localizing at 1q21^33^. In particular, they found that overexpression of CKS1B, a cell cycle regulator, was highly associated with an aggressive clinical course^34,35^ and was found to promote myeloma cell growth by activating cyclin-dependent kinases and through SKP2-mediated ubiquitination of the tumor suppressor p27^Kip1^ ^34,36^. Subsequently, CKS1B was later shown to activate myeloma cell growth through upregulation of the STAT3 and MEK/ERK pathways^37^.

MCL-1 is a member of the BCL2 family of anti-apoptosis proteins with a genetic locus at 1q21^38^. It has been well described that, with the exception of the t(11;14) MM subset, myeloma cells and normal plasma cells are highly dependent on MCL1 for survival^39,40^. Within CoMMpass, it was found that by RNA sequencing, higher expression of MCL1 was found as copy number of 1q increases^41^, and conversely, very high MCL1 gene expression highly correlates with presence of +1q among patients with newly diagnosed MM^42^. MCL-1 dependency is also enhanced by signaling from IL-6 within the bone marrow microenvironment^43^, and notably, the IL-6 receptor (IL-6R) is also encoded at 1q21. Soluble IL-6R levels rise with increasing copy number of 1q, but the impact on outcomes has been unclear^44–46^.

Recently, ADAR1 has been identified as a potentially important gene located at 1q21. ADAR1 is an RNA editing protein involved in A to I post-transcriptional modification, and overexpression leads to aberrant hyperediting and MM cell proliferation^47^. In CoMMpass, higher editing levels as mediated by ADAR1 were associated with inferior survival, independent of 1q copy number^48^. The P150 isoform of ADAR1 was subsequently found to promote oncogenesis through the STAT3 pathway, and this was further enhanced when combined with increased activity of IL-6R^49^.

PDZK1 is overexpressed in MM cell lines and drives resistance to chemotherapy in vitro^50^. Although this has been largely underexplored, a recent study showed that PDZ binding kinase is induced by IL-6 and promotes growth through STAT3 pathway^51^, yet another example of a protein at the 1q21 locus that is involved in the IL6-STAT3 continuum. PSMD4 and ILF2 have been implicated in resistance to specific treatments and are summarized later in the review.

## Outcomes

While +1q has been most extensively studied within the context of multiple myeloma, it has also been found in approximately 30% of patients with smoldering myeloma^22^. When detected by FISH or GEP-70 classification, +1q appears to be an independent risk factor for progression from SMM to MM^22,52,53^. The increased risk of progression to symptomatic myeloma is likely related to the association between +1q and genomic instability that has been described above, leading to additional mutational burden and progression to overt malignancy.

Prior to the widespread adoption of immunomodulatory drugs (IMiDs) and proteasome inhibitors (PIs) in the management of myeloma, the impact of +1q was evaluated in several clinical trials. In the IFM99-02 and IFM99-04 trials, induction with vincristine, doxorubicin, and dexamethasone (VAD) was evaluated prior to tandem autologous stem cell transplantation (ASCT)^54^. In these studies, gain(1q23) by comparative genome hybridization (CGH) and single nucleotide polymorphism (SNP) array was highly prognostic of early death on multivariate analysis^55,56^. Similarly, in the CMG2002 trial, patients with +1q21 by FISH who were treated with VAD induction had inferior outcomes than those without the abnormality^57^.

The Total Therapy 2 trial (TT2) performed by the UAMS group evaluated the implementation of thalidomide (in TT2) and other novel agents into the initial management strategy for patients along with chemotherapy induction, tandem transplantation, and consolidation/maintenance. In TT2, it was noted that patients with +1q at diagnosis had similar responses, but worse PFS and OS compared to those without +1q and ultimately derived no benefit from the addition of thalidomide^17^. In the Myeloma IX study, which also incorporated thalidomide into induction and maintenance for both transplant eligible and ineligible patients, +1q was also a poor prognostic factor^58,59^. When combined with adverse IgH translocations or del(17p), the impact of +1q was additive, and the effect was just as pronounced when found with hyperdiploid MM as with high-risk cytogenetics^60,61^.

Despite this early evidence suggesting inferior outcomes, a key meta-analysis raised questions about whether +1q was, in fact, an independent prognostic factor^25^. This study included three cohorts of patients—two from the Mayo clinic who were treated with high-dose therapy and ASCT, as well as the TT2 trial. In the analysis, 1q gain as determined by CKS1B copy number or the ratio of CKS1B to total chromosome 1 copies was again shown to be associated with high-risk clinical features, other high risk CAs, and poor survival. However, regardless of methodology to define +1q, when adjusted for other high-risk cytogenetic abnormalities, +1q was not found to be a significant prognostic factor on multivariate analysis. Subsequently, in an analysis of the GMMG-3 and GMMG-4 trials, that evaluated induction with doxorubicin/dexamethasone in combination with vincristine (VAD), thalidomide (TAD), or bortezomib (PAD) followed by transplantation, +1q21 was again found to be associated with high-risk disease but insignificant on multivariate analysis^62^. As such, although the IMWG has stated that +1q must be absent for a patient to qualify as standard risk^63^, +1q was not routinely evaluated at many centers for years and most Phase 3 clinical trials in newly diagnosed MM have not uniformly collected or reported data for +1q.

Several retrospective series have evaluated outcomes of patients with +1q21 myeloma after the implementation of PI/IMiD induction regimens. The Emory group found that among MM patients treated with lenalidomide, bortezomib, and dexamethasone (RVd) and risk-adapted maintenance among transplant-eligible patients, those with +1q by FISH had worse PFS and OS compared to those without +1q. Patients with amp(1q) had poor survival regardless of other cytogenetic abnormalities, however, there was a discrepancy among patients with gain.(1q) Patients with standard risk and gain(1q) had no significant impact on PFS compared to standard risk alone, whereas patients with gain(1q) plus t(4;14), t(14;16) or del(17p) had dismal PFS. Interestingly, patients with high risk cytogenetics but no +1q had outcomes similar to those with standard risk disease, suggesting a “double hit” effect^26^. In contrast, the Mayo group showed that OS was decreased among patients with +1q regardless of copy number, other cytogenetic abnormalities, and whether patients received a PI, IMiD, or both as a part of induction^64^. Two independent studies found that patients with chromosome 1 abnormalities, (C1A), including either +1q or del(1p), had inferior outcomes compared to those without C1A^65,66^. Outside of the United States, a study of outcomes within the Austrian myeloma registry, investigators found that the impact of +1q is dependent on copy number, but even gain(1q) was sufficient to abrogate the good prognosis of the hyperdiploid group defined by gains of 11q^67^.

Observational genomic analyses have shown similar results. In the Myeloma Genome Project, which collected comprehensive genomic data among 1273 patients with newly diagnosed myeloma, amp(1q) in combination with ISS stage 3 comprised a group of patients who had dismal outcomes, similar to those with the combination of del(17p) and mutated TP53^68^. In CoMMpass, PFS, and OS both decreased with each respective copy of 1q as determined by DNA sequencing^26^. Notably, although these retrospective and observational studies differ somewhat regarding methodology and the relative importance of copy number and other cytogenetic abnormalities, they were all conducted in the era of highly-active PI and IMiD induction therapy and the presence of +1q (or C1A) was found to be an independent prognostic factor for inferior outcomes, even when accounting for other CAs and clinical stage. As such, it appears that although outcomes for MM patients have improved with widespread implementation of PI/IMiD induction, patients with +1q have not benefitted as much, and clearly need to be evaluated in prospective clinical trials.

Two recent studies have reported data for +1q in newly diagnosed MM. The Myeloma XI trial, conducted in the UK, evaluated lenalidomide or thalidomide in combination with cyclophosphamide and dexamethasone (CRd and CTd, respectively), and later added an arm including carfilzomib with CRd (KCRd), followed by ASCT for eligible patients and maintenance with lenalidomide vs observation. In this study, patients with +1q had worse OS, and the prognostic effect was more prominent among those with amp(1q) or co-occurrence of additional high-risk cytogenetic abnormalities^69^. However, although +1q was an independent prognostic factor when evaluating cytogenetics alone, with the addition of the SKY92 GEP signature, even amp(1q) was no longer a significant factor, as nearly all of these patients were captured by the high-risk SKY92 signature. The FORTE study randomized transplant-eligible patients to receive 4 cycles of carfilzomib with cyclophosphamide and dexamethasone (KCd) followed by ASCT and 4 cycles of KCd consolidation, carfilzomib with lenalidomide/dexamethasone (KRd) for 4 cycles, followed by ASCT and 4 cycles of KRd consolidation, or 12 cycles of KRd without transplantation. In this study, patients with gain(1q) had worse survival in the KCd_ASCT and KRd12 arms, but the risk was abrogated in the KRd_ASCT arm. However, it was noted that patients with amp(1q) had dismal outcomes regardless of treatment arm, unless they were able to achieve negative minimal residual disease (MRD)^70^. More data regarding the outcomes of patients with and without +1q in Phase 3 clinical trials are compiled in Table 2.

**Table 2:** Outcomes of patients with +1q in randomized studies of newly diagnosed multiple myeloma.

Trial	Study treatments	N with 1q	Outcomes by 1q status
HOVON65/GMMG-HD4^71,112^	A: VAD → ASCT → mThal B: PAD → ASCT → mBort	Total +1q: 111 (32.3%) Gain(1q): 95 (27.6%) Amp(1q): 16 (4.7%)	96 month OS (+1q vs no +1q) Arm A: 28% vs 55% (*p* < 0.001) Arm B: 36% vs 62% (*p* = 0.006) OS HR by 1q copy number Gain(1q) vs normal 1q: 1.65 (*p* = 0.0010) Amp(1q) vs normal 1q: 2.48 (*p* = 0.0062)
TT2^17^*	A: no Thalidomide B: with Thalidomide	Total +1q: 205 (42.8%) Gain(1q): 117 (24%) Amp(1q): 88 (18%)	+1q vs normal 1q by treatment arm Arm A: 5 yr OS 55% vs 73% Arm B: 5 yr OS 49% vs 84% Outcomes by copy number Gain(1q) vs normal 1q: 5 yr OS 53% vs 78%, *p* < 0.001 Gain(1q) vs amp(1q): 5 yr OS 53% vs 50%, *p* = 0.453
Myeloma IX^60,69†^	A: CTD → ASCT B: CVAD → ASCT C: CTDa → mThal vs none D: MP → mThal vs none	Total +1q: 340 (39.1%)	+1q vs normal 1q Median PFS: 13.8mo vs 22.1mo; HR 1.53 (*p* = 6.7 × 10^−9^) Median OS: 31.0mo vs 54.8mo; HR 1.61 (*p* = 1.81 × 10^−8^)
Myeloma XI^69‡^	A: CTD → ASCT → mR vs none B: CRD → ASCT → mR vs none C: KCRD → ASCT → mR vs none D: CTDa → mR vs none E: CRDa → mR vs none	Total +1q: 357 (34.5%) Gain(1q): 277 (26.7%) Amp(1q): 80 (7.7%)	Gain(1q) vs normal 1q Median PFS: 21.8mo vs 30.1mo HR 1.56 (*p* = 3.53 × 10^−7^) 24mo OS: 77.5% vs 83.5% HR 1.67 (*p* = 3.30 × 10^−5^) Gain(1q) vs Amp(1q) Median PFS: 21.8 mo vs 19.4 mo; HR 0.91 (*p* = 0.54) 24 mo OS: 77.5% vs 63.8%; HR 1.36 (*p* = 0.09)
FORTE^70^	A: KCd → ASCT → R vs KR B: KRd → ASCT → R vs KR C: KRd12 → R vs KR	Total +1q: 181 (45.3%) Gain(1q): 129 (32.3%) Amp(1q): 52 (13.0%)	Gain(1q) vs normal 1q Median PFS: 53 mo vs NR; HR 1.65 (95% CI 1.14–2.37) 3 yr OS: 88% vs 94%; HR 1.88 (95% CI 0.98–3.58) Amp(1q) vs Gain(1q) Median PFS: 21.8 mo vs 53 mo; HR 1.84 (95% CI 1.21–2.81) 3 yr OS: 55% vs 88%; HR 1.84 (95% CI 1.73–5.68) Amp(1q) vs normal 1q Median PFS: 21.8 mo vs NR; HR 3.04 (95% CI 1.99–4.65) 3 yr OS: 55% vs 94%; HR 5.88 (95% CI 3.10–11.17)
S1211^101^	A: RVd → m. RVd B: Elo-RVd → m. Elo-RVd	Total +1q: 47 (47%)	Arm A vs Arm B Median PFS: 41 mo vs 32 mo; HR 0.761 (80% CI 0.459–1.261) Overall survival (all patients, multivariate analysis) +1q21 vs no 1q21: HR 0.776 (80% CI 0.388–1.552)

*The TT2 study reported all patients with chromosome 1 abnormalities as Amp1q21, but reported copy number separately.

^†^Patients assigned to intensive (randomized to A or B) or non-intensive (randomized to C or D) at discretion of investigator.

^‡^Patients were assigned to intensive (randomized to A or B; arm C added with a protocol amendment) or non-intensive (randomized to D or E) at discretion of investigator. If patients achieved only a partial or minimal response after induction, they were also randomized to intensification with VCD versus no intensification.

In summary, amp(1q) is uniformly associated with poor survival and should be considered a high risk cytogenetic abnormality. Although most studies suggest that gain(1q) is also high risk, its impact does not appear to be as detrimental as amp(1q), and the presence of other high risk cytogenetic abnormalities or gene expression profiles may be important factors in whether this abnormality imparts additional risk. In order to truly determine whether gain(1q) is, itself, a key prognostic marker or predictive of treatment outcomes, it will be essential for clinicians to evaluate for and document the presence or absence of an abnormality and copy number of 1q in all patients, and to report this data in a uniform matter, alongside other frequently reported cytogenetics such as IgH translocations, del(17p), and trisomies.

## Management implications

Ideally, standard management recommendations should be determined by prospective data from randomized controlled clinical trials. However, aside from the recent trials mentioned above, the standard of care in MM has evolved without much evidence regarding the relative benefit of novel combinations on patients with +1q. As such, clinicians are faced with the challenge of making treatment recommendations for a large population of patients with MM who are suggested to have inferior outcomes with the current standards of care, but no strong evidence to suggest benefit from alternative strategies.

It is generally believed that patients with high-risk disease derive benefit from proteasome inhibitors, particularly the t(4;14) and del(17p) subsets based on clinical data showing that bortezomib could abrogate the poor prognosis of these patients^71,72^. However, data among patients with +1q are conflicting, and as detailed below, several studies have suggested that +1q may actually confer resistance to bortezomib. In the Total Therapy 3 (TT3) trial, patients with +1q were noted to have early progression when treated with bortezomib compared to those without +1q, and GEP suggested that this may have been related to overexpression of PSMD4, a proteasome subunit encoded at 1q21 that was highly correlated with 1q copy number in TT3^73^. PSMD4 was identified as a poor prognostic factor in both the GEP-70 and GEP-80 models developed by UAMS-MIRT group, and PSMD4 overexpression appears to promote resistance to bortezomib, with increased resistance seen with additional copies of 1q21^73^. On the contrary, a recent study suggested that a high MCL1 gene signature, typically seen in patients with +1q, was predictive of longer PFS and OS when treated with bortezomib^42^. An, et al. found that patients with +1q had inferior outcomes with bortezomib-based induction, whereas it did not affect outcomes when thalidomide was used^74^. In the HOVON65/GMMG-4 trial, although patients treated with bortezomib had superior OS at 96 months compared to VAD, the outcome of patients with +1q was far inferior to those who did not have +1q. In the relapsed/refractory setting, several studies showed that patients with +1q had inferior outcomes with bortezomib-based regimens compared to patients without +1q^75–77^, although this was not a universal finding^78^. Preclinical data suggest that a NEDD8 inhibitor overcomes resistance to bortezomib among CKS1B overexpressing cells in vitro^79^, though this has not yet been translated to patient care.

It has been postulated that carfilzomib, a more potent proteasome inhibitor, may be able to overcome resistance to bortezomib among patients with high-risk myeloma. Pharmacogenomic analysis of patients treated with carfilzomib after prior bortezomib exposure in the TT3 trials and in Total Therapy 6 showed that expression of PSMD4, as well as other proteasome subunits, was increased in response to carfilzomib but not bortezomib, perhaps indicating that carfilzomib may be able to more effectively inhibit this and other subunits of the proteasome in MM, with increased gene expression reflecting the cell’s attempt to restore functionality of the inhibited proteasome subunit^80^. Early phase clinical trials have shown impressive response rates and MRD negativity when treated with carfilzomib-based induction, including high-risk patients^81,82^. However, the only Phase 3 data comparing RVd to KRd showed no difference in response rates or PFS between the two regimens among patients with standard risk myeloma or t(4;14)^83^. Outcomes for patients with +1q in this trial have not yet been reported. Although outcomes of patients treated with KRd induction and transplant in FORTE are encouraging and could be used to justify the use of KRd induction and consolidation with ASCT in transplant-eligible patients, it must be noted that no prospective, randomized trial has demonstrated superiority of KRd to VRd in newly diagnosed MM.

Regarding the utility of ASCT for patients with +1q MM, data are conflicting. A retrospective series from MD Anderson showed that patients with high CKS1B had inferior PFS following ASCT compared to those without this abnormality^84^. ILF2, a gene located at 1q21, is involved in homologous recombination in response to high-dose chemotherapy, and promotes myeloma cell resistance to DNA damaging agents such as melphalan^85^. However, in a CIBMTR analysis, +1q patients were not found to have inferior outcomes after ASCT^86^, and data from the FORTE trial suggest that patients with +1q who are treated with KRd induction have longer PFS when ASCT is employed compared to KRd alone. Based upon these data, we recommend upfront ASCT for patients with +1q who are transplant eligible.

The use of monoclonal antibodies (mAb) in the treatment of patients with MM has become ubiquitous, and based upon impressive data in the frontline setting by adding daratumumab to standard therapies^87–90^, many experts have recommended that a CD38 mAb be added to standard induction regimens. However, although daratumumab has clearly demonstrated efficacy among high-risk patients in the relapsed/refractory setting, the benefit of adding daratumumab in newly diagnosed high-risk patients has been more difficult to establish^91^. A recent meta-analysis showed that, with pooled data, there is a benefit to adding daratumumab to induction therapy for high-risk patients, though not as substantial as among standard risk^92^. Additionally, the majority of this benefit was driven by the MAIA study, when daratumumab was added to a doublet, lenalidomide/dexamethasone, as opposed to PI/IMiD triplets in the other studies. Notably, data regarding the impact of CD38 mAbs in patients with +1q are extremely limited. In one retrospective series, +1q21 or GEP-70 high risk score portended poor prognosis among relapsed/refractory patients treated with daratumumab^93^. In the ICARIA-MM study, patients with +1q21 by FISH who were treated with isatuximab plus pomalidomide/dexamethasone (Pd) had superior PFS compared to those treated with Pd alone^94^, although patients with +1q21 were noted to have inferior ORR and PFS in both treatment groups compared to those without 1q. Recently, the JAK-STAT pathway has been implicated in downregulation of CD38 on the surface of MM cells^95^. If STAT3 overactivation is driven by +1q21, this could potentially explain a mechanism for resistance to daratumumab, though this has not been directly assessed.

There are no data reporting the specific impact of consolidation or maintenance therapy for patients with +1q MM. Lenalidomide maintenance until progression is recommended for most patients in the United States based upon the PFS benefit seen in the FIRST trial for transplant-ineligible patients^96^, and an overall survival benefit seen in the CALGB 100104 study^97^ and a pivotal meta-analysis of post-transplant maintenance^98^. However, patients with high risk disease do not derive as much benefit from this approach^99^, and alternative strategies for these patients have been explored. Bortezomib maintenance has been suggested to be beneficial among high-risk patients^71,99^, but the impact of bortezomib on outcomes of patients with +1q was not directly assessed in these studies. The Emory group has demonstrated PFS and OS rates far superior to historical controls among patients with high risk MM with the use of RVd maintenance after transplant, but +1q was not assessed in that study^100^. In a prospective study, the SWOG S1211 study, which exclusively enrolled patients with high-risk MM and employed VRd with or without elotuzumab (Elo) as induction and maintenance, showed no benefit for the addition of Elo, but demonstrated a median PFS for the RVd arm of 33.5 months, which outperformed the expected PFS for this group and is postulated to be due to prolonged PI/IMiD maintenance^101^. Recent data presented from the FORTE study suggests that carfilzomib plus lenalidomide maintenance improves PFS compared to lenalidomide monotherapy as post-transplant maintenance among all patients, including those with high-risk disease, further supporting the use of PI/IMiD maintenance in the post-transplant setting in high risk MM.

Given the plethora of potential drivers at 1q21, it is enticing to investigate the potential to target some of these genes and pathways. Particularly given the success of the BCL2 inhibitor, venetoclax, in other lymphoid malignancies and in generating impressive responses in the t(11;14) subset of MM^102^, many attempts have been made to target MCL1 in MM. Preclinical data suggest that patients with +1q may be less likely to be resistant to MCL1 inhibitors^41^. However, because MCL1 is a very labile protein and since the intrinsic apoptosis pathway has substantial complexity in its regulation, this approach has been largely unsuccessful. Recently, several small molecule MCL-1 inhibitors have been developed and are in early phase clinical trials^103–105^.

FcHR5 is a surface protein of unknown function that is highly expressed on the surface of MM cells and has a genetic locus in the chromosomal breakpoint at 1q21. Preclinical studies have demonstrated that FcHR5 is expressed at higher levels among patients with MM harboring +1q21 compared to those without +1q21^106^. Cevostamab is a bispecific antibody targeting FcHR5 and has shown promising activity in Phase 1 studies^107^. Although data among patients with +1q21 are unknown in that study, it will be interesting to see whether these patients have increased sensitivity to targeting by cevostamab. Targeting of CKS1B has also been investigated and is in early stages of development^108^. Based upon the common pathway of STAT3 activation of candidate genes at 1q21, this may also be an intriguing approach for patients with +1q MM.

**Fig. 1:**
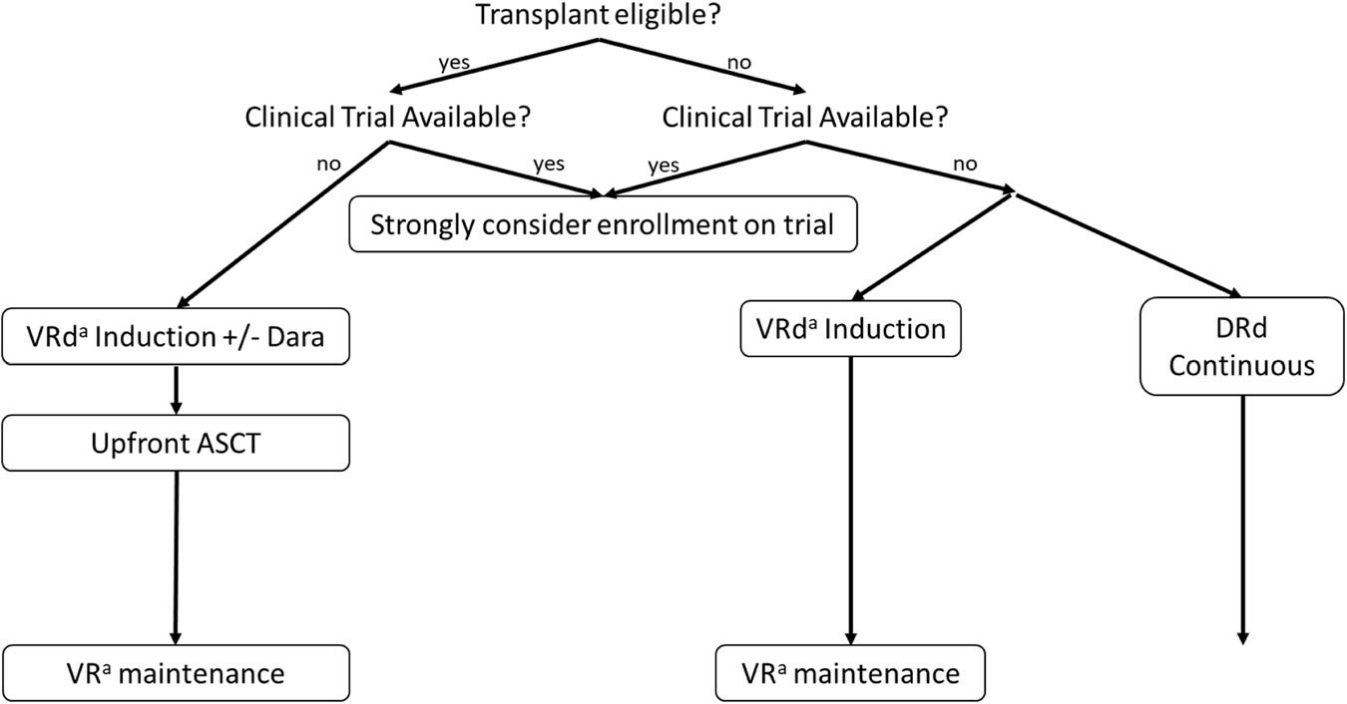
Proposed management recommendations for patients with newly diagnosed multiple myeloma and +1q21 by FISH. V = bortezomib; R or Len = lenalidomide; d = dexamethasone; Dara or D = daratumumab; ASCT = autologous stem cell transplantation with high-dose melphalan conditioning; HR CA = high risk cytogenetic abnormalities. ^a^Carfilzomib may be substituted for bortezomib if neuropathy is present.

Improving outcomes and defining the optimal management strategy for patients with high-risk myeloma is one of the most important questions faced by myeloma researchers today. Risk-adapted treatment strategies have been reported^109^ and are becoming more frequently utilized, as the “one-size-fits-all” approach to the management of MM is becoming outdated. Furthermore, the implementation of advanced genomics and dynamic risk assessment based on MRD testing is likely to improve our ability to identify patients at risk of early relapse who are in need of alternate treatment strategies^110^. However, at this time cytogenetics remain the most widely utilized genomic factor in risk stratification of MM, and the importance of obtaining accurate cytogenetic information at diagnosis cannot be understated in order to identify patients who may need risk-adapted approaches. Ultimately, despite the frequency with which +1q is identified and its association with inferior outcomes, the optimal management of patients with +1q MM is poorly defined, largely due to the wide variability in assessment and reporting of 1q abnormalities. Based upon the data covered in this review, our general approach to management of newly diagnosed MM patients with +1q is summarized in Fig. 1.

## Conclusion

Outcomes for patients with high-risk MM, including those with +1q, remain suboptimal and investigational approaches are recommended. As more effective treatment options including adaptive cellular therapy, bispecific antibodies, and targeted therapy continue to be developed and are implemented in the management of MM, these will likely lead to improved outcomes for patients with high-risk MM including those with +1q. Because these novel therapies have almost always shown to be effective for all subsets, but to a lesser degree in high risk patients, determining the optimal time point to utilize these treatment strategies will be of critical importance to improve survival and potentially cure patients. Clinical trials such as S1211^101^ and MASTER^111^ that selectively enroll high-risk patients are critically needed, and patients with +1q, particularly those with amp(1q), should be considered for enrollment in these trials. On the contrary, due to the profound efficacy of highly-active treatment options, some patients may not require such an aggressive approach, and could be considered for de-escalation of therapy. It will be critically important to determine whether +1q is a determining factor in predicting outcomes with these risk-adapted strategies, and it is essential that these abnormalities, identified in 30–40% of newly diagnosed MM patients, be documented and studied uniformly. Based upon the exciting progress in therapeutics for MM, optimism is clearly warranted for the exciting possibilities of long-term survival outcomes of patients with MM, including those with +1q. Hope abounds that with proper risk stratification and potential targeted approaches, these patients can experience prolonged survival with excellent quality of life for many years.
